# openEyeTrack - A high speed multi-threaded eye tracker for head-fixed applications

**DOI:** 10.21105/joss.01631

**Published:** 2019-10-23

**Authors:** Jorge Paolo Casas, Chandramouli Chandrasekaran

**Affiliations:** 1 Department of Biomedical Engineering, Boston University, Boston, MA 02215, USA; 2 Department of Anatomy and Neurobiology, Boston University, Boston, MA 02118, USA; 3 Department of Psychological and Brain Sciences, Boston University, Boston, MA 02215, USA; 4 Center for Systems Neuroscience, Boston University, Boston, MA 02215, USA

## Statement of Need

When faced with a decision, an organism uses information gathered by its senses in order to determine the best course of action. Vision is one of the primary senses, and tracking eye gaze can offer insight into the cues that affect decision-making behavior. Thus, to study decision-making and other cognitive processes, it is fundamentally necessary to track eye position accurately. However, commercial eye trackers are often very expensive, and incorporate proprietary software to detect the movement of the eye. Closed source solutions limit the researcher’s ability to be fully informed regarding the algorithms used to track the eye and to incorporate modifications tailored to their needs. Here, we present our software solution, *openEyeTrack*, a low-cost, high-speed, low-latency, open-source video-based eye tracker. Video-based eye trackers can perform nearly as well as classical scleral search coil methods and are suitable for most applications (Kimmel, Mammo, & Newsome, 2012). High-speed eye trackers improve ability to detect saccades and microsaccades for real-time behavioral and physiological experiments and also improve eye position estimation.

### Planned Use Cases:

We expect to incorporate *openEyeTrack* in research concerning the neural dynamics of cognition, decision-making, and motor-control conducted at the Chandrasekaran Lab and in labs performing human psychophysics experiments at Boston University. We also anticipate performing additional tests of *openEyeTrack* in experiments at the Zimmerman Lab at the University of Minnesota. These experiments will further validate *openEyeTrack*, help identify necessary enhancements, and provide additional performance metrics.

## Software and Hardware components

*openEyeTrack* is a video-based eye-tracker that takes advantage of OpenCV (Bradski & Kaehler, 2016; OpenCV, 2019a, 2019b), a low-cost, high-speed infrared camera and GigE-V APIs for Linux provided by Teledyne DALSA (Teledyne DALSA, 2018), the graphical user interface toolkit QT5 (The Qt Company Ltd., 2013) and cvui, the OpenCV based GUI (Bevilacqua, 2018). All of the software components are freely available. The only costs are from the hardware components such as the camera (Genie Nano M640 NIR, Teledyne DALSA, ~$450, ~730 frames per second) and infrared light source, an articulated arm to position the camera (Manfrotto: $130), a computer with one or more gigabit network interface cards, and a power over ethernet switch to power and receive data from the camera.

By using the GigE-V Framework to capture the frames from the DALSA camera and the OpenCV simple blob detector, *openEyeTrack* can accurately estimate the position and area of the pupil. We include pupil size calculations because of its putative link to arousal levels and emotions of the subject (Võ et al., 2008).

## Multithreading provides improvements over existing open source solutions

*openEyeTrack* is modeled on other open-source eye trackers currently available such as *“Oculomatic”* (Zimmermann, Vazquez, Glimcher, Pesaran, & Louie, 2016). However, most of these programs are single-threaded: the frames are captured, analyzed, and displayed sequentially, only executing the next stage once the previous stage completes its processing. Although single-threaded methods have become more effective over the years, these stages are time-consuming and can limit the overall performance. In order to increase performance, *openEyeTrack* was developed as a multithreaded application. The capture, display, data transmission, and most importantly, pupil detection components all happen within their separate threads. By incorporating multiple threads, the processing speed of the frames can match the frame capture rate of the camera, allowing for the lossless processing of data.

## Algorithm

**Figure 1:**
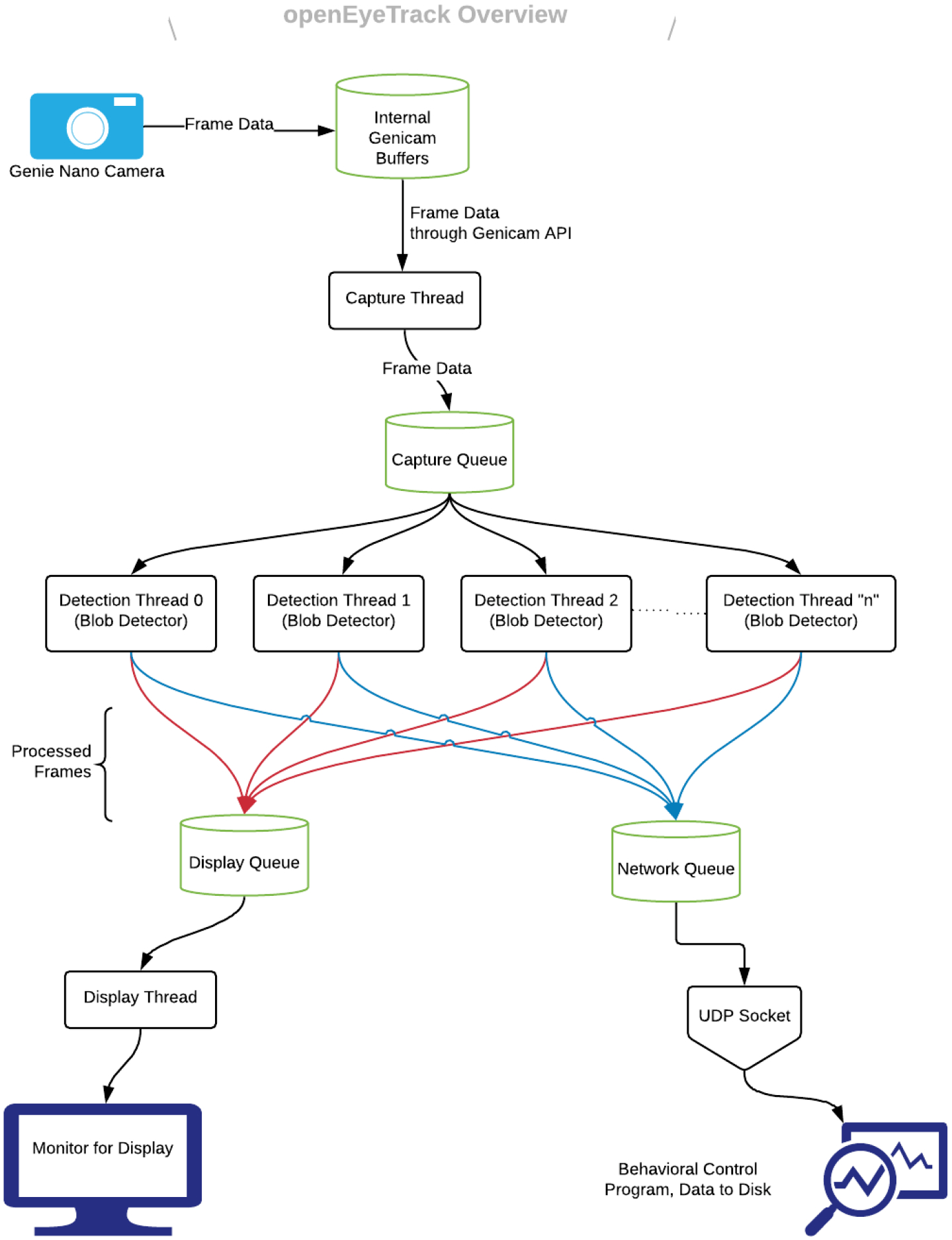
A visual depiction of the overall software and hardware architecture in openEyeTrack.

Figure 1 shows the workflow for *openEyeTrack*. As frames transition between the capture, detection, and display stages, they are stored in queues which enable the separate stages to run independently and allow for asynchronous capture, detection, and display. Once the camera grabs a new frame, it is briefly stored in the Genicam memory buffers before being extracted and packaged by the “capture thread” into a struct and stored in a queue. This approach ensures that the sequence of acquisition frames is preserved and that the frame acquisition process can occur without being slowed down by the pupil detection process or display related processes. The frames in the “capture queue” are then popped off by the “n” (user-specified) detection thread(s). Each “detection thread” takes the data from the “capture queue,” converts it into an OpenCV Mat object, applies the OpenCV blob detection algorithm to identify the pupil, and notes the key features. Each detection thread also outputs the eye position (i.e., center of the pupil) as text on the frame and draws a circle around the blob identified as the pupil. All of these steps are very time consuming, which is why we recommend initializing multiple threads for higher performance. Once the detection stage has completed, the frames are stored in a “display queue” that the “display thread” will grab from to show the images. The detection threads also package the frame and keypoints information into a struct object and stores it in a “network queue.” The “network thread” reads from this queue and sends out packets over a UDP socket for downstream applications.

## Performance

Under the conditions at the time of development, we were able to achieve frame acquisition rates of up to 715 frames per sec (fps) and display rates of up to 145 fps. Although more threads, in theory, should increase speed, four detection threads were sufficient to keep up with the camera. Performance was significantly improved when we used the tool provided by Teledyne Dalsa that adjusts various features of the network buffers and allows higher throughput transmission from the camera to the computer. Additionally, environmental lighting significantly affects the speed at which the blob detection occurs. The OpenCV blob detector by default looks for black blobs, and thus more light allows for easier detection by increasing the contrast between darker and lighter areas of the image. To facilitate blob detection, we also apply binary thresholding to the images. The user can also specify a region of interest for the blob detector, which again improves processing time. For eye tracking, it is necessary to have an infrared IR light source to obtain a eye image with increased contrast between the pupil and the surrounding regions.

## Limitations

Our eye tracking solution is not meant to solve all gaze tracking issues which may be more readily addressed in commercial solutions.

1. *openEyeTrack* cannot be used if the head is freely moving. In our approach, which only detects the pupil, head motion is confounded with pupil motion. One future solution is to use both the corneal reflection and the pupil to allow for head-free eye tracking. We anticipate implementing corneal reflections in future versions of *openEyeTrack*.
2. *openEyeTrack* does not output signals to analog channels which is a typical feature of commercial eye trackers. These analog signals were proxies for the analog signals from scleral search coils used for eye tracking.
3. Using *openEyeTrack* requires knowledge of Linux and some degree of comfort with the command line to compile and install various components — it is not as seamless and polished as commercial solutions. On the other hand, it provides open-source code for eye-tracking.

*openEyeTrack* is available on GitHub at https://github.com/chand-lab/openEyeTrack. A more detailed description of usage can be found under the README.md and USAGE.md files located in the repository.
